# Odorants for Surveillance and Control of the Asian Citrus Psyllid (*Diaphorina citri*)

**DOI:** 10.1371/journal.pone.0109236

**Published:** 2014-10-27

**Authors:** Iliano V. Coutinho-Abreu, Lisa Forster, Tom Guda, Anandasankar Ray

**Affiliations:** 1 Department of Entomology, University of California Riverside, Riverside, California, United States of America; 2 Center for Disease Vector Research, University of California Riverside, Riverside, California, United States of America; Monell Chemical Senses Center, United States of America

## Abstract

**Background:**

The Asian Citrus Psyllid (ACP), *Diaphorina citri*, can transmit the bacterium *Candidatus* Liberibacter while feeding on citrus flush shoots. This bacterium causes Huanglongbing (HLB), a major disease of citrus cultivation worldwide necessitating the development of new tools for ACP surveillance and control. The olfactory system of ACP is sensitive to variety of odorants released by citrus plants and offers an opportunity to develop new attractants and repellents.

**Results:**

In this study, we performed single-unit electrophysiology to identify odorants that are strong activators, inhibitors, and prolonged activators of ACP odorant receptor neurons (ORNs). We identified a suite of odorants that activated the ORNs with high specificity and sensitivity, which may be useful in eliciting behavior such as attraction. In separate experiments, we also identified odorants that evoked prolonged ORN responses and antagonistic odorants able to suppress neuronal responses to activators, both of which can be useful in lowering attraction to hosts. In field trials, we tested the electrophysiologically identified activating odorants and identified a 3-odor blend that enhances trap catches by ∼230%.

**Conclusion:**

These findings provide a set of odorants that can be used to develop affordable and safe odor-based surveillance and masking strategies for this dangerous pest insect.

## Background

The Asian Citrus Psyllid (ACP), *Diaphorina citri* (Hemiptera: Psyllidae), is attracted to the young flush of citrus plants where it feeds on the sap as well as uses as a site for mating, oviposition, and development of the nymphs [1], [2]. ACP is a vector of *Candidatus* Liberibacter bacteria the causative agent of Huanglongbing (HLB), also known as citrus greening disease, a major threat to citrus cultivation worldwide [3], [4]. Management of HLB relies mostly on insecticide spraying and removal of infected trees [4], however the emergence of insecticide resistance [5] and the potential of abandoned citrus groves as reservoirs of HLB [6] pose a significant threat to the commercially managed groves.

Other psyllid species transmit viruses and bacteria to other economically important cultivars as well, such as carrot, pear, and apple [4], [7]. Interestingly, some psyllids can shift hosts seasonally [7]. For instance, in the winter, the carrot psyllid *Trioza apicalis* migrates from carrot plants to conifers. Interestingly, both plants display similar volatile chemical profiles [8], suggesting that the psyllid olfactory system may sense both hosts.

Like the other members of the suborder Sternorrhyncha (Hemiptera), psyllids have a relatively simple olfactory system [9], [10]: the antennae are covered with a small number of trichoid and pit-like placode sensilla (rhinarial plates, RPs) [9]–[11]; and the antennal lobes are devoid of defined glomeruli [12]. The rhinarial plates are known as the principal odorant sensors [13], containing plant volatile–sensing olfactory neurons [9], [14]. In laboratory settings, ACP has been shown to be attracted to odors release by citrus flush shoots [15], mildly attracted to an odorant released by infected citrus trees [16], and repelled by sulfur-containing compounds released by guava leaves [17] and garlic cloves [18]. These studies point to the feasibility of developing an odorant-based approach for improving ACP surveillance and control.

Recently we carried out a comprehensive analysis of odor detection by the ACP rhinarial plates (RPs) using single-sensillum electrophysiology and a panel of 119 odors and compared odor coding to that of *Drosophila melanogaster* and *Anopheles gambiae* [19]. Here we identify which odorants from this panel are detected by ACP at lower concentrations and show that some activating odorants can potentially be used as attractants. In addition we identify inhibitors that can be used to block detection of citrus volatiles. In behavioral experiments, we identify a blend of three odorants that increases attraction of ACP to traps in field settings.

## Results and Discussion

ACPs are highly invasive insects, which are rapidly spreading to different parts of the world [3]. Despite their importance, effective tools for surveillance are not currently available. Identifying volatiles that evoke ACP Odorant Receptor Neuron (ORN) responses can lead to the identification of odorants to be used as tools for ACP surveillance and control.

**Figure 1:**
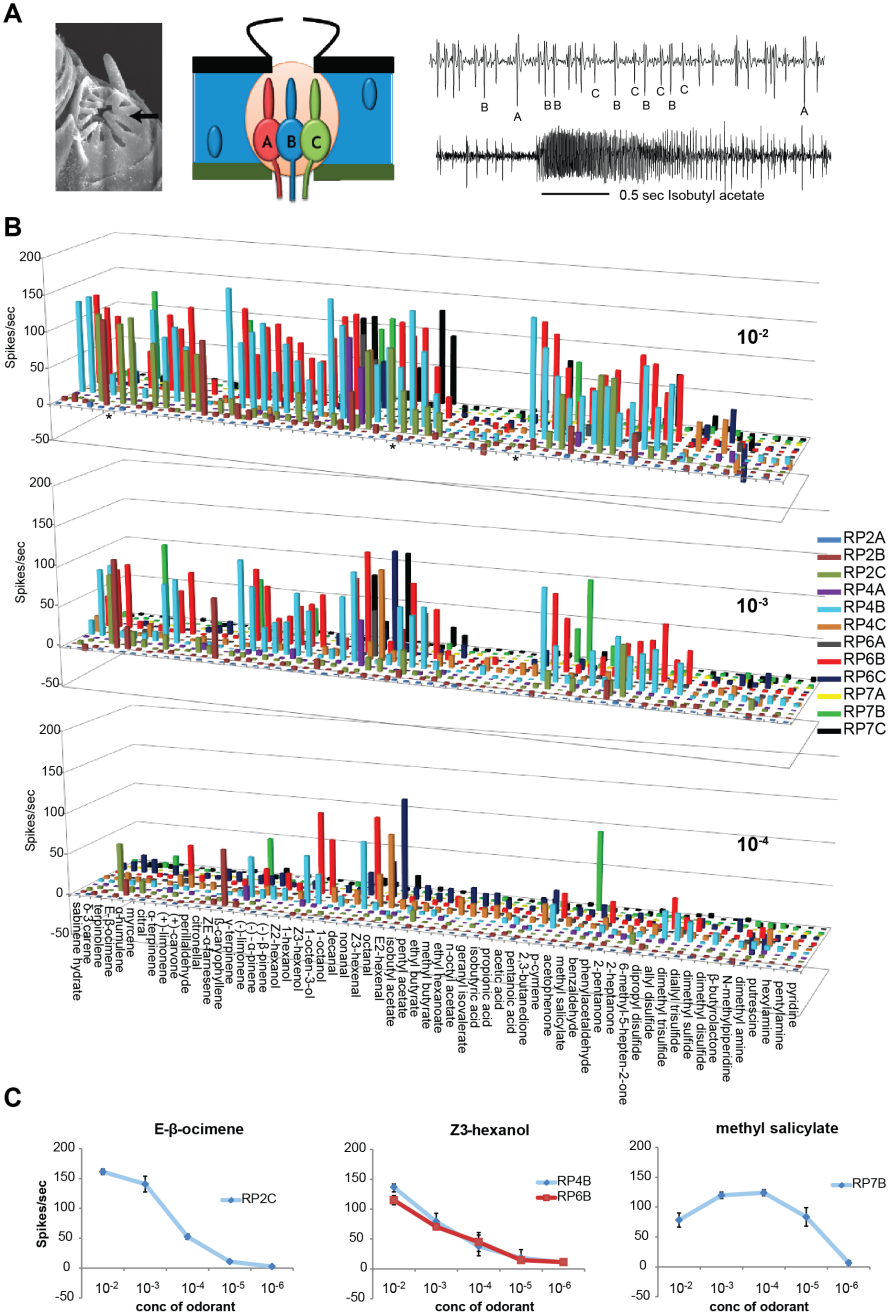
ACP rhinarial plate and odor sensitivity. (**a**) Left: Scanning electron micrograph of a rhinarial plate sensillum (arrow). Middle: Schematic of rhinarial plate cross section, showing three ORNs housed in a pit-like structure. Right: Representative action potential traces from a RP4 sensillum showing (top) spontaneous activity with spike amplitudes A, B, and C neurons marked, and (bottom) during a 0.5 sec stimulus with isobutyl acetate (10^−2^) indicated with a line. (**b**) Graphs depicting mean ORN responses to 61 odorants that activate and/or inhibit at least one rhinarial plate ORN at 10^−2^, 10^−3^, and 10^−4^ dilution (spikes/sec). N = 3 per stimulus. (**c**) Dose-response curves. Chemical volatiles were screened at five different concentrations (10^−2^ to 10^−6^) against specific RP-ORNs. n = 3. Error bars: SEM.

Psyllids are likely to be exposed to a range of odor concentrations during their flight towards a citrus tree. The ACP olfactory system is likely to encounter odors at very low concentrations when it is far away. Plant odors are detected by pit-like placodea sensilla on the ACP antenna, also known as rhinarial plates. Each RP houses three odorant receptor neurons (Fig. 1a; [19]). Odorants that are able to activate ACP rhinarial plate ORNs at low concentrations may be candidates for long-range attractive cues. In order to identify these odorants, we performed a dose-response analysis using odorants that we had previously identified as activators and tested them at lower concentrations. We found that the intensity of ORN responses varied considerably across odor concentrations, decreasing in breadth at lower concentrations (Fig. 1b, Table S1). When odorants were tested at 10-fold lower concentration (0.1%) than the one initially tested (1%), ∼42% of the ORNs evoked responses (Fig. 1b, Table S1). At this concentration, only α-humulene, γ-terpinene, nonanal, octanal, *p*-cymene, and methyl salicylate induced strong responses (≥100 spikes/sec). Most ORNs (except RP2A and RP7A) displayed at least one strong activator at this concentration. When odorant concentrations were reduced by 100-fold (0.01%) from the initially tested concentration, only seven odorants evoked robust responses, which indicates that the ACP antennae are more sensitive to these plant volatiles (Fig. 1b, Table S1).

We performed additional dose-response experiments with selected strong activators to identify the most sensitively detected activators for several neurons (Fig. 1c). The RP7B neuron showed the highest sensitivity of any ORN: it detected methyl salicylate at concentrations as low as 10^−5^ (Fig. 1c). It has been reported that methyl salicylate is released by citrus trees that are infested with ACP and is mildly attractive in laboratory assays [16].

**Figure 2:**
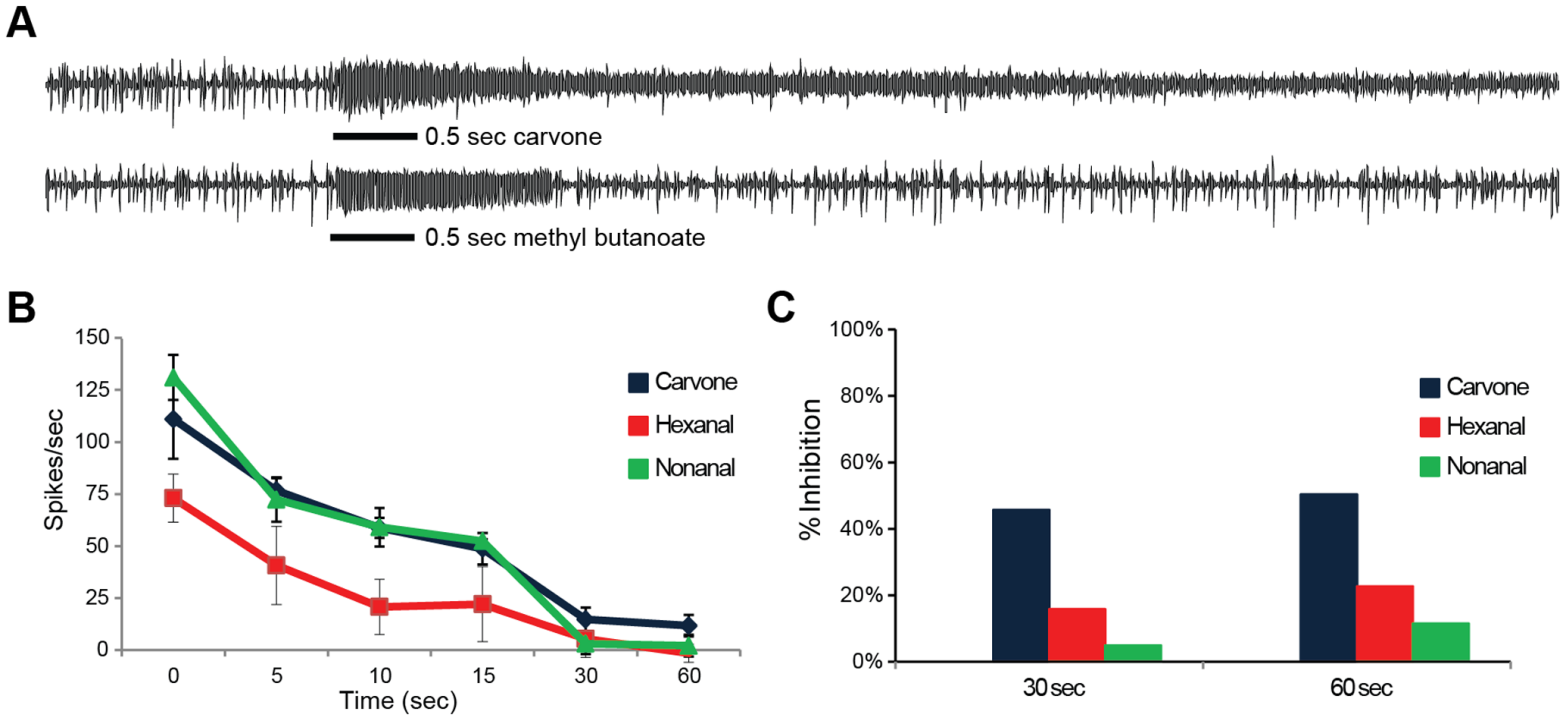
Prolonged activators of RP-ORNs. (**a**) Representative traces of tonic response to carvone (top) and phasic response to methyl butanoate (bottom) upon 0.5 sec odor stimulation (bar). (**b**) Mean activity of RP4B neuron at times after an initial 3 sec stimulus of indicated odorant (10^−1^). (**c**) Percentage inhibition derived from the ratios of acetophenone responses upon prolonged-activator stimulation over acetophenone responses upon solvent delivery at the same time points. Black bar: 0.5 sec stimuli duration. n = 3.

Among odorants that activate ACP rhinarial plate ORNs, a few induced tonic responses lasting beyond the stimulus duration (Fig. 2a). Since prolonged activators disrupt ORNs from efficiently reporting fluctuating odor concentrations along an odor plume boundary [20], [21], they have the potential to mask citrus plant volatiles from ACP. In order to test for prolonged activation, the strong activators were tested at a higher concentration (10^−1^). We identified some which evoked prolonged-activation for up to 30 sec after stimulus delivery (Fig. 2b). A brief exposure to such odorants, especially (+)-carvone, was able to mask subsequent detection of pulses of acetophenone by nearly 50% up to 30 sec after the initial exposure (Fig. 2c).

**Figure 3:**
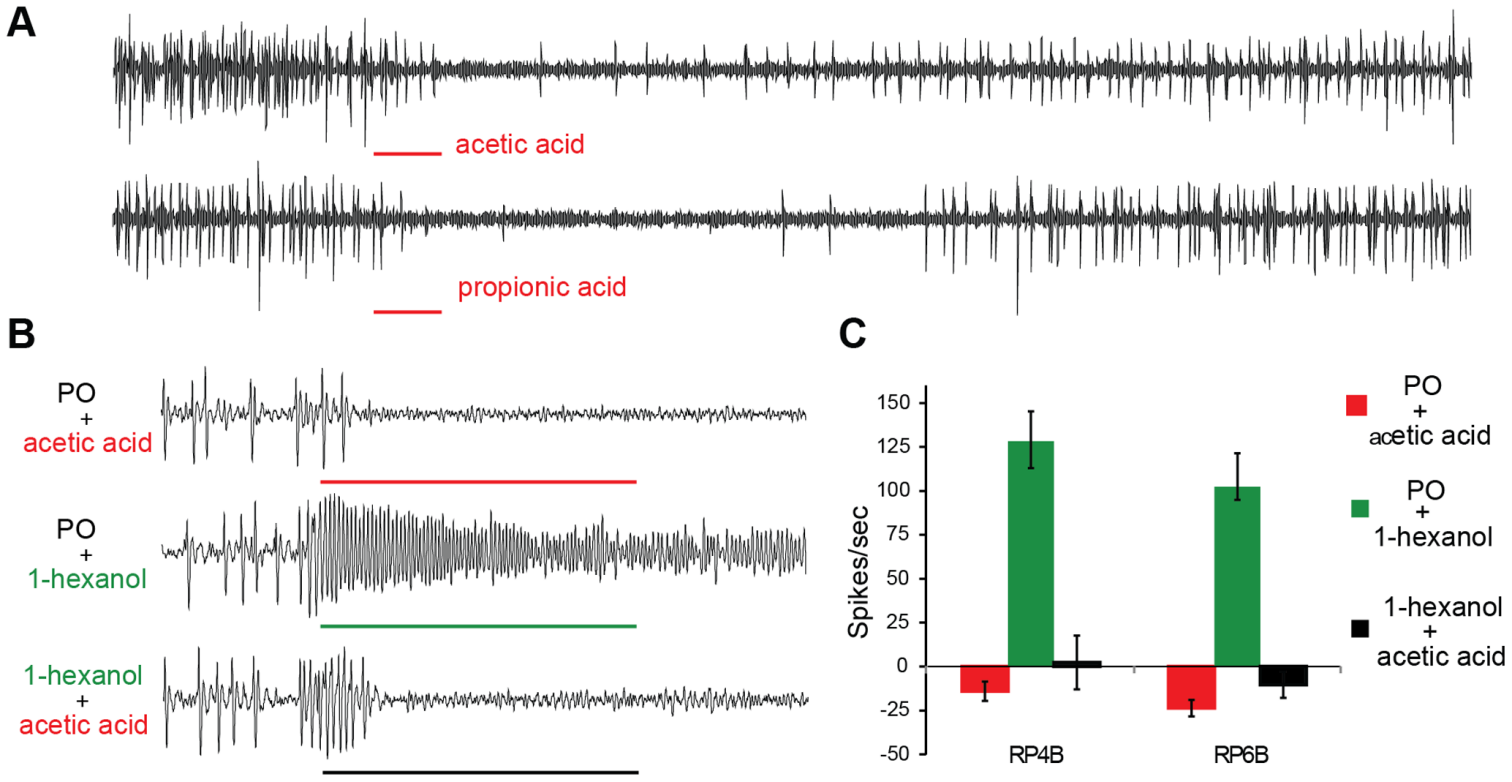
Inhibitors of RP-ORNs. (**a**) Representative traces displaying inhibition of spontaneous activity of RP4B to 0.5 sec stimulus with either acetic acid or propionic acid. (**b**) Representative traces of RP6 to overlapping stimuli of acetic acid (10^−1^) with solvent (PO) or 1-hexanol (10^−2^). (**c**) Mean responses of RP4B and RP6B responses to treatments as in (b). Black bar: 0.5 sec stimuli duration. PO, paraffin oil. n = 3.

Odorants that inhibit ORN activity can also mask detection of citrus volatiles. A number of odorants in our panel inhibited ORN spontaneous activity by >50% (Table S1). Amongst them, acetic acid and propionic acid blocked spontaneous activity for several seconds beyond the duration of the stimulus application (Fig. 3a). In order to determine if these inhibitors are able to suppress odor-induced activation of ORNs, we simultaneously exposed the ACP antenna to a strong activator and an inhibitor. Remarkably, acetic acid completely suppresses RP4B and RP6B ORN activation by 1-hexanol, a strong plant-associated activator (Figs. 3b and c). This degree of inhibition is unusual amongst insect ORNs and has only been observed for Gr–expressing neurons that detect CO_2_ [22]–[24]. Not only is acetic acid a strong inhibitor, but also inexpensive and safe for use around plants and therefore has potential to mask host-plant volatile detection.

One of the major gaps in ACP control is the lack of effective surveillance traps to track the rapid spread of these highly invasive insects that are rapidly spreading globally [3]. In order to test whether the odors we identified in this electrophysiology analysis as activators of ACP ORNs are effective as attractants, we performed field trials to test whether activating odorants can increase the efficiency of commonly used blunder yellow sticky traps. Since agricultural orchards with ACP are quarantined, destroyed, or heavily sprayed with insecticides [4], trials were performed in an urban area of El Monte, California, USA, where we had access to ACP-infested citrus trees. From preliminary field trials using single-compound lures of octanal, nonanal, β-caryophyllene, methyl salicylate, p-cymene, acetophenone, myrcene, ethyl butyrate, p-cymene, and blended lures at two different concentrations (data not shown), we were able to identify the most promising attractant as a 3-odor blend (myrcene, ethyl butyrate, and p-cymene) for further experimentation. Herbivorous insects are often attracted to blends of volatiles released by host plants [25]–[27]. The three odorants of this attractive blend strongly stimulate the RP4B and RP6B ORNs and the RP2C and RP7C more moderately at 10^−2^ dilution (Table S1). The RP4B and RP6B are broadly activated by several of the same volatiles released by citrus plants, suggesting that they may play a role in attraction behavior.

**Figure 4:**
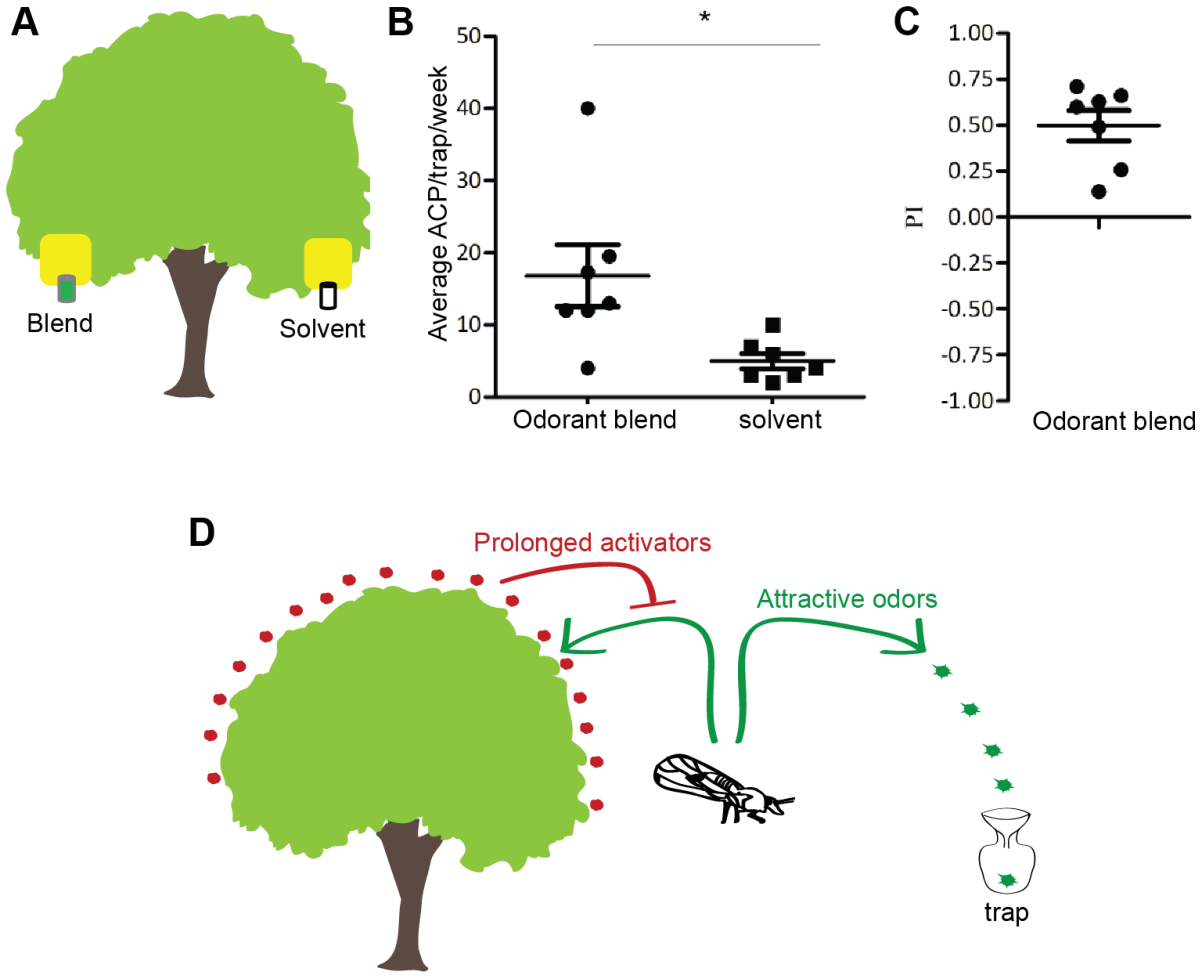
Identification of an odor lure in field trials. (**a**) Schematic of assay with a yellow sticky trap holding 3-odor blend lured trap and solvent trap on contra-lateral side. (**b**) Mean number of ACPs caught per trap per week (*, p = 0.01, n = 7 weeks, paired t-test; Data normally distributed, p>0.10, Kolmogorov-Smirnov test). (**c**) Mean preference index of ACP on 3-odor blend lured traps. n = 7. (**d**) Schematic representing the integrative Push and Pull strategy for ACP. Prolonged activators can potentially mask odor-mediated attraction of ACP to citrus trees. Additionally, ACP can be lured away from citrus by attractive odorants released by traps set elsewhere.

We next performed a more comprehensive field trial with the 3-odor blend spread over several weeks and found that the odor-lured yellow traps caught significantly more ACP per tree per week (16.8±4.28) as compared to solvent-control yellow traps placed on the same tree (5.0±1.07) (Fig. 4a,b,c, Table S2). This represented a ∼230% increase in trap catches and a preference index (PI) of 0.50±0.08 in the odor-lured traps (Fig. 4b, Fig. S1). These three chemicals are affordable, useful in small quantities, and reasonably safe for human handling suggesting that they could be of immediate utility in monitoring and surveillance.

## Conclusion

Using a combination of neurophysiology and behavior, we have identified a suite of odorants that are detected by the ACP olfactory system, some of which we show can modify the behavior of ACP and can potentially be used to develop tools to tackle its spread worldwide that causes millions of dollars of damage to crops. The toolkit includes prolonged activators and inhibitors that can be tested for repellency, an attractive odor blend, and several additional strong ORN activators that can be tested as lures. These odorants can be utilized in an integrated approach for ACP based on masking attraction (prolonged activators) and pull (activating odor lures) (Fig. 4d) [28]. Similar odorant-based approach can be taken to develop behavioral control strategies for other insect pests as well, which affect nearly a third of the world's food supply, and whose control programs are in desperate need for new generations of attractants and repellents [29].

## Materials and Methods

### Psyllid rearing

ACP (Texas strain) was reared at the Quarantine facility at the University of California, Riverside in 40×40×40 cm wood cages. ACP was fed on curry (*Bergera koenegii*) and citrus (*Citrus volkameriana*) plant (10–15 cm high) at a 3∶1 curry to citrus ratio. Rooms were maintained at 25±1°C and 45% relative humidity.

### Scanning electron microscopy

Scanning electron micrograph was taken as described in [19].

### Odor panel composition

Odor panel description is provided elsewhere [19]. Among the activators and inhibitors, 70% are FDA approved for human use and are likely to be safe to be deployed in control strategies against ACP (Table S3). We have also tested odors released by flush shoots of citrus plants, the mating, oviposition, and developmental site for ACP [15].

### Electrophysiology

Single-sensillum recording was performed as previously described [20], [23] with minor modifications outlined in [19]. For the longer prolonged activator assays, cartridges were prepared by placing odorants onto filter paper (200 µl, 10^−1^ dilution) placed into a 10 ml serological pipette through which air stimulus was blown. For inhibition (dual-delivery) assays, a controlled air pulse (0.5 sec; 10 ml/sec) was split by a Y connector between two cartridges and delivered into the same hole on the airstream tube by polypropylene tubes (10 cm) connected to the cartridges. Activators were applied into cartridges at 10^−2^ (1-hexanol) dilution whereas inhibitors were loaded at 10^−1^ dilution. Fifty microliters of each odor were used.

### Field trial

An odor blend composed of three chemical volatiles at 5% dilution in paraffin oil (myrcene, ethyl butyrate, and p-cymene) was deployed in citrus trees located in private land (backyards) in residential neighborhood in El Monte (CA, USA) that had been assigned to us by the California Department of Food and Agriculture (CDFA) after they obtained permission from the landowners for setting up traps. The test trees were located at 34°03′22.7″N 118°02′01.8″W, 34°02′28.4″N 118°01′38.3″W, and 34°02′32.7″N 118°01′36.3″W. The chemicals tested as lures were approved for field use by the Office of Environmental Health Hazard Assessment, California Environmental Protection Agency. To the best of our knowledge protected or endangered were not affected by our field study due to the limited number of traps set up each day. The chemicals for single-compound lures of octanal, nonanal, β-caryophyllene, methyl salicylate, p-cymene, acetophenone, myrcene, ethyl butyrate, p-cymene were chosen based on their ability to activate different RP-ORN combinations. The blend components broadly activate four ORNs, without activating the RP7B ORN. RP7B is activated strongly by methyl salicylate, an odor that induces ACP repellence at high concentrations [16]. Each odor was individually diluted to 5% in paraffin oil, 2 ml was loaded into glass vials (1 Dram; ≈3.7 ml), and the vials were kept inside a zipper seal sample bag (7×5 cm, Fisher Scientific). A bubble straw (1 cm diameter, 8 cm length) was vertically inserted into the bag so as to deliver the odors to the outside. Plastic bags were stapled at the base of Yellow sticky traps (Fig. S1). Odor-baited and solvent-baited traps were set up on the southwest and northeast sides of the trees. Traps were replaced and rotated every week (n = 7 weeks). Kolmogorov-Smirnov test was used to assess if number of caught psyllids were normally distributed, and paired t-test was carried out to assess whether the number of caught psyllids differences by blend-baited and solvent-baited traps were statistically significant. Preference index was calculated using the equation: PI = (#blend - #control)/(#blend+#control), where # is the average number of psyllids caught per treatment.

## Supporting Information

Figure S1 **Trapping device.** (**a**) Double-faced Yellow sticky trap attached to the blend delivery device. This device consisted of three glass vials within a sample bag. Odors are delivered to the outside by a bubble straw (2/3 inside and 1/3 length outside plastic bag). (**b**) Representative traps retrieved from citrus trees after one week trapping. Trap on the left was baited with solvent whereas the one on the right was baited with the three-odor blend. Caught psyllids are circled and marked by red dots.
(PDF)

Table S1 **RP-ORN responses of 61 activators and inhibitors across concentrations.** Left, responses to odorants delivered at 10^−2^ dilution (modified from [19]). Middle, responses to odorants delivered at 10^−3^ dilution. Right, responses evoked by odors delivered at 10^−4^ dilution. Chemical classes are color-coded. Responses to odor are shown in spikes per seconds and are subtracted from the spontaneous activity. Activations are labeled in yellow (≥50 spikes/sec). Inhibitory responses are highlighted in red (inhibition ≥50% of spontaneous activity).
(XLS)

Table S2 **Field trial.** Number of psyllids caught per tree per week, average number of psyllids caught per week, average preference index per week, and trial average preference index are shown. Date refers to the day each trap was set up each week. Low participation (>5 psyllids/tree in both traps) were excluded from analysis and are not included in the table. From March 1^st^ to March 29^th^, trapping was only carried out on tree EL11. From April 19^th^ to May 10^th^, trees EL3, EL10, and EL11 were subjected to trapping. Due to heavy rain in week of April 5^th^, April 12^th^, and April 26^th^, trapping was not performed.
(XLS)

Table S3 **Organoleptic properties of 61 activators and inhibitors (10^−2^).** Odorant common name, IUPAC nomenclature, Chemical class, CAS number (CAS #), Odor type, Odor strength, Odor description, Vapor pressure, and FDA regulation are shown. Sources: The Good Scents Company (www.thegoodscentscompany.com); PubChem (pubchem.ncbi.nlm.nih.gov); ChemSpider (www.chemspider.com); Sigma (www.sigmaaldrich.com). * FDA permits: FDA PART 172 (food additives permitted for direct addition to food for human consumption); FDA PART 173 (secondary direct food additive permitted in food for human consumption); FDA PART 175 (indirect food additives: adhesives and components of coating); FDA PART 176 (indirect food additives: paper and paperboard components); FDA PART 177 (indirect food additives: polymers); FDA PART 178 (indirect food additives: adjuvants, production aids, and sanitizers); FDA PART 182 (Substances generally recognized as safe); FDA PART 182 (indirect food additives: polymers); FDA PART 184 (direct food substances affirmed as generally recognized as safe). # No information available.
(XLS)
