# Supplementary figures and images for: Odorants for Surveillance and Control of the Asian Citrus Psyllid (Diaphorina citri)
Source: PLoS One. 2014 Oct 27;9(10):e109236. doi: 10.1371/journal.pone.0109236 (PMC4209970; doi:10.1371/journal.pone.0109236)

**A**

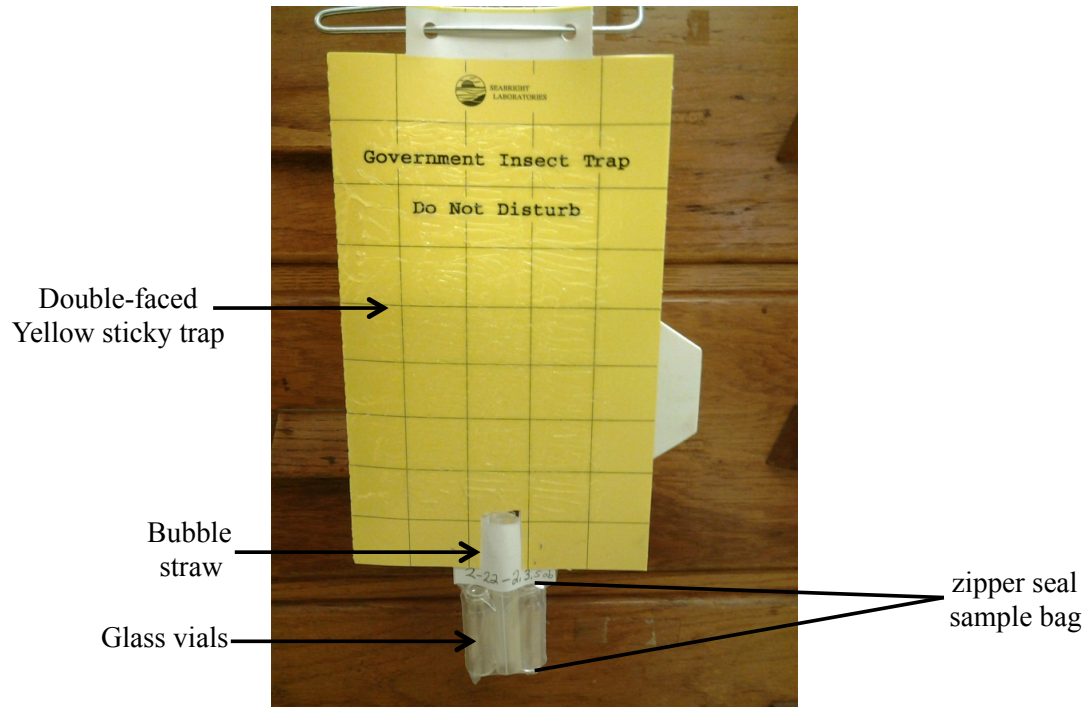

**B**

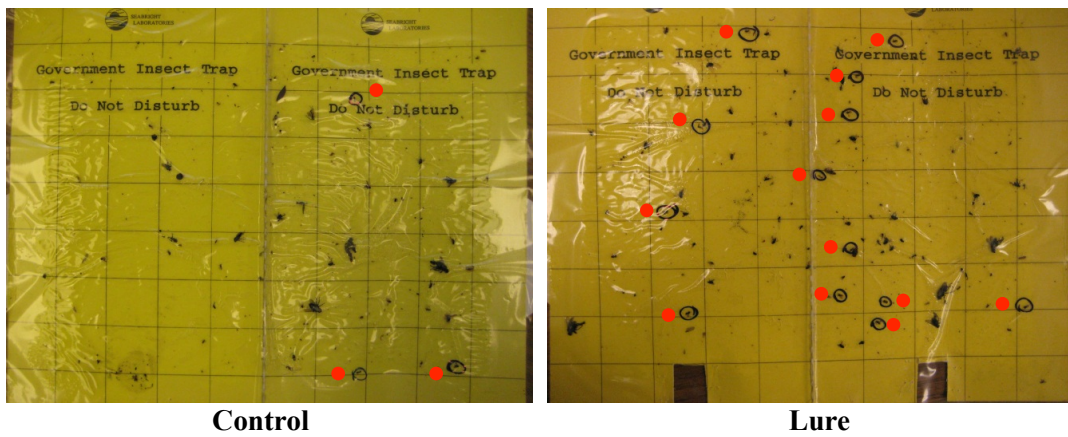

**Figure S1**

Supplement: Figure S1 — Trapping device. (a) Double-faced Yellow sticky trap attached to the blend delivery device. This device consisted of three glass vials within a sample bag. Odors are delivered to the outside by a bubble straw (2/3 inside and 1/3 length outside plastic bag). (b) Representative traps retrieved from citrus trees after one week trapping. Trap on the left was baited with solvent whereas the one on the right was baited with the three-odor blend. Caught psyllids are circled and marked by red dots. (PDF) [file pone.0109236.s001.pdf]
